# Evaluation of Serum Nitric Oxide Level in Patients with Oral Lichen Planus

**Published:** 2014-06

**Authors:** M. Mehdipour, A. Taghavi Zenouz, A. Bahramian, N. Gholizadeh, M. Boorghani

**Affiliations:** a Dept. of Oral Medicine, School of Dentistry, Shahid Beheshti University of Medical Sciences, Tehran, Iran.; b Dept. of Oral Medicine, School of Dentistry, Tabriz University of Medical Sciences, Tabriz, Iran.; c Dept. of Oral Medicine, School of Dentistry, Tehran University of Medical Sciences, Tehran, Iran.

**Keywords:** Lichen planus, Nitric oxide, Oxidative stress

## Abstract

**Statement of Problem:** Oral lichen planus (OLP) is a chronic inflammatory oral mucosal disease with indefinite etiology. In recent researches, free radicals have been deliberated as the possible etiology of inflammatory and autoimmune diseases.

Purpose: This study was aimed to evaluate the stress oxidative status with the nitric oxide (NO) index in a sample of Iranian population.

**Materials and Method:** In this descriptive-comparative study, serum NO level was assessed in 20 OLP patients as the case group and 20 healthy individuals as the control group. Collected data were analyzed by adopting two Sample t-test, using SPSS 16 software. The statistical significance level was set at *p*< 0.05.

**Results:** The mean serum NO levels in OLP patients and healthy controls were 17.1±3.4 ng/ml and 14.5±2.7 ng/ml respectively; which revealed a significant statistical difference (*p*= 0.009).

**Conclusion:** The results of the current study with its limitation may support the premise that higher serum levels of NO in patients with OLP might activate the process of lymphocytes and cellular immunity system; hence, possibly endorsing the effect of serum NO in pathogenesis of lichen planus.

## Introduction

Oral lichen planus (OLP) is a chronic inflammatory oral mucosal disease with unidentified etiology. It appears as white striations, papules, plaques, erythema, erosions or ulcers in the mouth and mostly affects the buccal mucosa, tongue and gingiva. Approximately, 0.005 percent of Australian adults develop intra-oral SCC each year [1].

Recently, free radicals are concerned for their role in inflammatory and autoimmune diseases. Various reactive oxygen species (ROS) are produced by different cells such as keratinocytes, fibroblasts, and inflammatory cells. Overproduction of these species may perhaps be a sign of cellular damage and destruction of anti-oxidative immune mechanisms. The imbalance between the systemic presentation of reactive oxygen intermediates and a biological system's ability to detoxify the reactive species easily; or else to repair the resultant damage; is described as the oxidative stress [2]. Overproduction of ROS can be a crucial mediator of damage to the cell structures, including lipids, membrane, proteins and DNA [3].

It should be noted that in the histopathologic appearance of lichen planus, basal cell degeneration, infiltration of inflammatory cells (like T lymphocytes), and destruction of keratinocytes are observed [1]. Free radicals and the other active oxygen compounds aggravate the inflammatory responses with the participation of T lymphocytes and destruct the lipid membrane of keratinocytes [4-5].

A perceptible increase of lipid peroxidation products and reduced antioxidant defensive system was reported in a study enrolled on the patients with genital lichen planus [2]. The authors pointed out the effect of stress oxidative indices in the pathophysiologic changes occurred in the basal cells of epidermis and they verified a decreased antioxidant defense and an increased oxidative damage to the lipids, proteins and DNA in LP [2].

The role of stress oxidative indices has been introduced in many autoimmune and inflammatory diseases such as atopic dermatitis, psoriasis vulgaris, vitiligo, and dermal lichen planus [6-9].

Since the etiology of lichen planus is still unidentified, the treatment of the disease would be a symptomatic approach. Most of the previous studies in this field have been carried out on the dermal and genital lichen planus; therefore, this study aimed to evaluate the stress oxidative status with the nitric oxide index in a sample of Iranian population with OLP to detect the etiopathogenesis of the disease and to give some intimation to find a relevant treatment. 

## Materials and Method

This descriptive-comparative study was approved by ethics committee of Tabriz Medical University (No: 9166). 20 patients with OLP, confirmed by clinical or clinicopathological diagnosis and 20 healthy individuals (as the control group) were selected from the local residents referring to the department of oral medicine, faculty of dentistry, Tabriz University of Medical Sciences to be recruited in this study. A clinical diagnosis of OLP was established when reticular or popular textures were present clinically, and in the histopathologic appearance; basal cell degeneration, infiltration of inflammatory cells like T lymphocytes should have been observed. The patients with 18 to 60 years old, diagnosed with oral lichen planus, were recruited in this study. The diagnosis of the keratotic lichen planus and the erosive lichen planus was confirmed by the clinical features and clinicopathological features respectively. Exclusion criteria were considered to be the presence of any stimulus leading to the lichenoid reactions including assumption of any medications; the appearance of the lesions near the amalgam restorations, presence of any factors which could alter the equilibrium of production and elimination of free radicals; (such as cigarette smoking, alcohol consumption), use of hydrogen peroxide mouth-rinse, having a diet full of fruit and vegetables, immunosuppressed patients, the use of antioxidant drugs (vitamin E and vitamin C), steroids, NSAIDS, and possible history of trauma or surgery during the last four weeks. Patients with systemic diseases, malignancies or dermal diseases which would influence the immune system were also excluded [2, 5].

The patients’ records were completed and necessary examinations were performed. The biopsy was then taken after procurement of a written informed consent. Then, 5 cc blood samples were taken from both groups and of the serum were isolated. The sera samples were kept at -70°C for one month. The experiment was conducted at laboratories of Imam-Reza Hospital, Tabriz University of Medical Sciences.

The measurement of NO serum level was carried out by employing Human NO ELISA kit (Cusabio; USA) and ELISA apparatus (ELISA micro plate reader; BIOTEK, Winooski, VT). The collected data were analyzed by Two-Sample t-test using SPSS 16 statistical software. *p*< 0.05 was deliberated as the statistical significance in this study. A proper post-hoc test was also engaged in the incidence of having significant differences.

## Results

**Figure 1:**
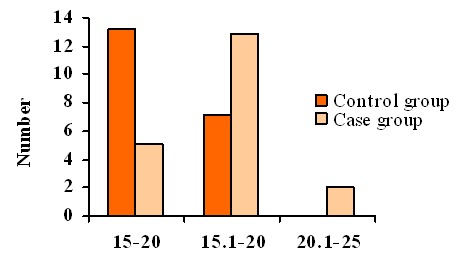
Histogram of NO serum levels in oral lichen planus patients and healthy control group according to staging.

The mean age of the OLP patients and the control individuals were 34.1 and 35.6 years old, respectively. The mean age of all participants was 34.8 years old. The mean serum NO levels in lichen planus patients and healthy individuals were (17.1±3.4) and (14.5±2.7 ng/ml), respectively. Two-Sample t-test revealed statistically significant difference in serum NO level between two groups (*p*= 0.009; Figure 1).

## Discussion

In the current study, the mean serum NO levels in patients with OLP was more than the control healthy individuals. In the earlier studies, increasing serum NO (an active free radical) has been found as a critical destructive mediator to the cell structures, including lipids, proteins and DNA which would clinically manifest as oral ulcers [3].This procedure justifies the formation of ulcer and erosion in the erosive lichen planus. Moreover, NO can provoke T4 lymphocytes and inhibit their apoptosis by regulating the potential of mitochondrial membrane [10]. Bogdan et al. [11] confirmed the role of NO as a regulator of development, differentiation and function of T and B lymphocytes.

NO has been reported to increase the T4 lymphocyte proliferation selectively [12]. Most aggregated lymphocytes in the lamina propria samples of patients with lichen planus is T4 lymphocytes [13-15]. Eversole et al. [16] concluded the presence of a combination of T4 and T8 lymphocytes in lichen planus ulcer; which, in turn, determines the clinical behavior of the disease.

The findings of the present study are comparable with the results of most of the previous studies that evaluated the stress oxidative status in biopsies or salivary samples of the patients with dermal or oral lichen planus. 

The findings yielded by this study are similar to the study of Sunitha et al. [17]; which reported the increased levels of salivary NO compared to the healthy individuals. In another survey in Turkey; Sezer et al. [9] studied 40 dermal lichen planus patients (9 individuals with reticular lichen planus) and reported a noticeable increase of NO in the affected group. With assessing the salivary levels of NO byproducts in 21 patients with oral lichen planus and 18 patients with recurrent aphtus, Ohashi et al.’s study concluded that NO increased significantly in the two patient groups [18]. In the study of Ergun et al. [19], salivary and serum level of malondialdehyde (MDA) and total anti-oxidative activity in 21 patients with OLP was investigated and revealed a significant statistical difference with healthy group. The findings of the current study were also in line with the results of their study.

In another study conducted on 45 patients with lichen planus and 45 control individuals in Egypt, the serum level of NO was substantially increased similar to the finding of our study [5]. Moreover, Agha Hoseini et al. [20] stated the oxidative stress processes play a role in OLP etiopathogenesis and reported an elevated level of MDA of saliva in OLP as an index of oxidative stress.

The findings of the present study differ from the results of study of Brennan et al. [21], in which the inducible form of NO synthase (one of the isoenzymes of nitric oxide synthase) was investigated in 30 patients with oral reticular lichen planus using immunohistochemistry. Their research failed to validate a significant difference in NO synthetize staining between the patients and the healthy control individuals [21]. This dissimilarity could be attributed to the different study methods since in our study, both reticular and erosive lichen planus cases were evaluated, whereas only reticular type was assessed in the Brennan et al. study. Moreover, in their study, biopsy samples (NO level at tissue samples) were used, in which the use of formalin could have caused false-negative results. The demographic differences in the evaluated samples may have also influenced these contradicting conclusions.

In the present study, only two forms of oral lichen planus were evaluated, therefore, the authors would suggest studying the problem of stress oxidative in different forms of oral lichen planus to confirm the opinion hypothesized on etiopathogenesis of the disease. Moreover, the authors believe that the antioxidant drugs can possibly change the increased oxidative status in OLP, even though theoretically. This concept apparently needs more investigations and corresponding animal studies. 

## Conclusion

The characteristics of NO in the activation process of lymphocytes and cellular immune system is very similar to the pathogenesis of lichen planus .Therefore nitric oxide is suspected to cause erosion and ulceration as a result of cell damage. Regarding its role in the lichen planus pathogenesis, antioxidant drugs could probably be considered in the treatment of OLP. Further studies need to be performed on the larger samples to realize the role of the nitric oxide and other oxidative stress indices in oral mucosal diseases thoroughly.
